# Cascade of Denoising and Mapping Neural Networks for MRI R2* Relaxometry of Iron-Loaded Liver

**DOI:** 10.3390/bioengineering10020209

**Published:** 2023-02-04

**Authors:** Qiqi Lu, Changqing Wang, Zifeng Lian, Xinyuan Zhang, Wei Yang, Qianjin Feng, Yanqiu Feng

**Affiliations:** 1 School of Biomedical Engineering, Southern Medical University, Guangzhou 510000, China; 2 Guangdong Provincial Key Laboratory of Medical Image Processing, Southern Medical University, Guangzhou 510000, China; 3 Guangdong Province Engineering Laboratory for Medical Imaging and Diagnostic Technology, Southern Medical University, Guangzhou 510000, China; 4 Guangdong-Hong Kong-Macao Greater Bay Area Center for Brain Science and Brain-Inspired Intelligence & Key Laboratory of Mental Health of the Ministry of Education, Southern Medical University, Guangzhou 510000, China; 5 School of Biomedical Engineering, Anhui Medical University, Hefei 230000, China

**Keywords:** MR relaxometry, R2* mapping, liver iron overload, convolutional neural network, cascade network

## Abstract

MRI of effective transverse relaxation rate (R2*) measurement is a reliable method for liver iron concentration quantification. However, R2* mapping can be degraded by noise, especially in the case of iron overload. This study aimed to develop a deep learning method for MRI R2* relaxometry of an iron-loaded liver using a two-stage cascaded neural network. The proposed method, named CadamNet, combines two convolutional neural networks separately designed for image denoising and parameter mapping into a cascade framework, and the physics-based R2* decay model was incorporated in training the mapping network to enforce data consistency further. CadamNet was trained using simulated liver data with Rician noise, which was constructed from clinical liver data. The performance of CadamNet was quantitatively evaluated on simulated data with varying noise levels as well as clinical liver data and compared with the single-stage parameter mapping network (MappingNet) and two conventional model-based R2* mapping methods. CadamNet consistently achieved high-quality R2* maps and outperformed MappingNet at varying noise levels. Compared with conventional R2* mapping methods, CadamNet yielded R2* maps with lower errors, higher quality, and substantially increased efficiency. In conclusion, the proposed CadamNet enables accurate and efficient iron-loaded liver R2* mapping, especially in the presence of severe noise.

## 1. Introduction

Primary hemochromatosis (i.e., hereditary hemochromatosis) and secondary hemochromatosis (e.g., thalassemia, sickle cell disease, chronic liver disease, and long-term red blood cell transfusions) result in excessive iron deposition in the body and cause structural and functional damage to vital organs, especially the liver [1]. Accurate quantification of hepatic iron concentration (HIC) is critical to managing and monitoring iron chelation therapy. Magnetic resonance imaging (MRI) is commonly adopted for hepatic iron quantification instead of liver biopsy, which is invasive and has a low inter- and intra-observer repeatability [2,3]. MRI effective transverse relaxation rate (R2* = 1/T2*) of liver parenchyma linearly rises with HIC and has been established as a reliable and accurate quantitative measure of HIC in transfusion-dependent patients [4].

Due to the high scan efficiency [5] and high intercenter agreement [6], the MRI R2* technique is widely performed for assessing HIC in clinical practice. A representative R2* value is typically obtained for the liver parenchyma and then transformed to HIC. Compared with regions of interest (ROI)-based R2* quantification, which fits averaged decay signals in ROI, R2* mapping has the ability to depict the spatial distribution of HIC [7]. The R2* map is obtained by pixel-wise fitting multiple images at varying echo times (TEs) to an exponential decay function using a multi-gradient recalled echo (mGRE) sequence. However, R2* mapping is adversely affected by the presence of fat in tissue and noise-related bias. Fat-water oscillations can be eliminated by using fat-suppression techniques [8]. The noise level in MRI is usually quantified in terms of a signal-to-noise ratio (SNR), a measure of signal strength relative to background noise. In scenarios of severe liver iron overload and high magnetic field (for example, 3.0 T), the intensities of signals in the acquired serial T2*-weighted images usually decay and thus have low SNRs [9]. Noise in T2*-weighted magnitude MR images, which can be characterized by Rician or non-central chi distribution [10], degrades the accuracy and precision of R2* estimation and thus might mislead clinical interpretation. It is essential to suppress the effect of noise on R2* quantification for assessing HIC.

The effect of Rician noise includes signal bias and fluctuation. The offset model [4] represents the signal bias by adding a constant to the mono-exponential function. In theory, the bias caused by Rician noise depends on the intensity of the underlying truth signal. Thus, the offset model cannot correctly address the effect of Rician noise on the decay signal, and it tends to overestimate R2* by a factor depending on the noise level [11]. The truncation model only fits data with high signal intensities (i.e., SNRs) to minimize the impact of noise [12]. However, this method tends to underestimate high R2* where the signal decays rapidly, and the SNR of remaining data after truncation is still poor [11]. The first-moment (M^1^NCM) and second-moment (M^2^NCM) noise correction models can correctly describe the effect of Rician noise on the R2* decay signal by fitting the measured data to the first and second moments of mono-exponential signal in the presence of Rician noise, respectively [11]. The impact of intensity fluctuations on R2* mapping can be reduced by filtering images before curve fitting [13]. Recently, a curve fitting method with adaptive neighborhood regularization (PCANR) [14] was developed to improve liver R2* mapping by incorporating noise suppression and curve fitting into a unified regularization framework. This approach can avoid the error propagation from image filtering to parameter mapping and generate R2* maps with significantly reduced noise and well-preserved tiny structures. Although the above methods achieve high performance in noise correction, the slow speed of computation is a shortcoming of them and needs to be improved.

Recently, deep learning has been successfully applied to MR parameter mapping. Quantitative parameter maps can be estimated from measured images through deep neural networks. The popular U-Net has shown inspiring results in the parameter mapping domain [15,16,17,18,19,20]. The performance of deep learning-based parameter mapping can be further improved by incorporating regularization terms, such as total variation [16], or incorporating data consistency terms, such as MR physical models [16,17,18,19], into the loss function during network training. Although the training of deep networks for parameter reconstruction is computationally intensive, the implementation of a trained network is highly efficient. However, deep neural networks are highly nonlinear and nonconcave, and they may yield unstable results for parameter reconstruction, especially in the presence of perturbations, such as noise [21]. Actually, noise in MR images can also be suppressed by using deep learning methods [22,23,24]. As such, we hypothesize that combined image denoising and parameter mapping using deep learning would benefit the R2* mapping of an iron-loaded liver, especially in the presence of severe noise for high HIC.

In this paper, we propose a novel two-stage cascade network named CadamNet for MRI R2* mapping of an iron-loaded liver. The proposed CadamNet directly reconstructs R2* map from multi-echo T2*-weighted images with Rician noise. CadamNet accomplishes R2* mapping task in two stages, including the image denoising stage and the parameter estimation stage, which are achieved through a denoising network and a parameter mapping network, respectively. An analytical biophysical model is incorporated into the training of the parameter mapping network to enforce data and model consistency. A mass of simulated data generated from clinical liver data is used to train the networks, the construction of which is guided by a biophysical model. Extensive evaluation experiments are conducted on simulated and clinical liver data. The proposed method achieves accurate and efficient R2* mapping of an iron-loaded liver, especially in the presence of severe noise.

## 2. Materials and Methods

### 2.1. Clinical Datasets

For clinical data, we retrospectively selected 121 patients with thalassemia major (53 females, 68 males, aged 9–66 years) with iron overload from normal to severe according to their R2* measurements. All MRI examinations were performed using a 1.5 T Siemens Sonata MRI scanner (Siemens Medical Solutions, Erlangen, Germany) and a 6-channel anterior array coil combined with a 2-channel spine array coil. Axial T2*-weighted images were acquired using a 2D spoiled gradient-echo sequence with the following parameters: repetition time = 200 ms, number of echoes = 12, TE_min_ = 0.93 ms, echo spacing = 1.34 ms, flip angle = 20°, slice thickness = 10 mm, signal average = 1, acquisition matrix = 64 × 128, and in-plane resolution = 3.125 × 3.125 mm^2^. Fat saturation was used to eliminate fat-water oscillations [8]. The multiple TE images were acquired within a breath-hold of approximately 13 s. The study was approved by the local institutional review board, and informed content was obtained. In total, 121 clinical datasets with a size of 64 × 128 × 12 were acquired. The 12 channels contain data acquired at 12 TEs, respectively. One hundred datasets were used to generate simulation data for training the proposed method, and twenty-one datasets were used to generate simulation data for testing and also used for in vivo assessment.

### 2.2. Simulation Datasets

Generally, the training of deep networks is conducted in a supervised fashion, which needs to collect input images and corresponding ground-truth labels. However, high-quality ground-truth R2* maps are unavailable due to the low SNR of iron-loaded liver data. Thus, we used simulated data to train the network. The simulation data were generated from clinical data to ensure that they reflected structural features in real liver data. The clinical data were first denoised using the non-local means method [25] to reduce the influence of noise on the quantification of the relaxation parameters. The signal intensity at zero TE (S_0_) and R2* maps were then calculated from the denoised clinical data using the automatic truncation method [26] with the same truncation rules. These calculated R2* maps were used as the reference R2* maps of the simulation data. After that, noise-free T2*-weighted images with TEs similar to that in the clinical data acquisition were synthesized by using the mono-exponential model with parameters from the calculated S_0_ and R2* maps. Finally, the simulated images at multiple TEs were added with Rician noise with varying levels. The liver parenchyma R2* values of training data were approximately uniformly distributed over a wide range of 45 to 956 s^−1^. The training data were augmented by rotating and flipping the input data to satisfy the requirement of a large number of training samples. In addition, overlapping patches were extracted from the samples via a fixed sliding step of eight [27]. A total of 15,200 multi-channel 2D patches with a size of 32 × 32 × 12 were generated for training. The resolution of the simulated data was the same as the clinical data.

### 2.3. CadamNet Framework

**Figure 1:**
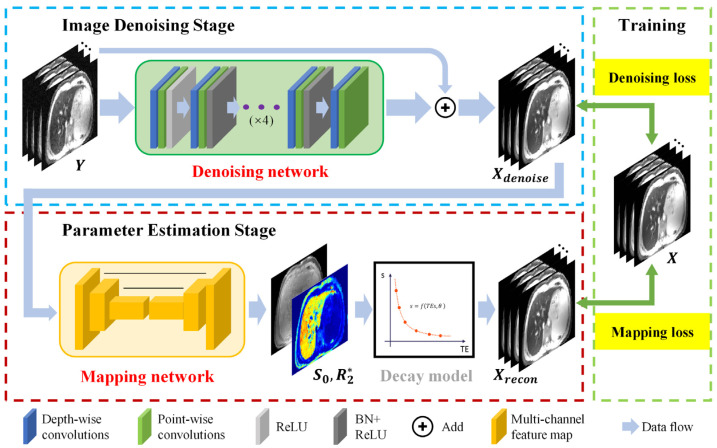
Overall architecture of the proposed CadamNet framework. Blue box: image denoising stage, the inputs to the denoising network are 12 images (12 echo times). The outputs are 12 denoised images. Red box: parameter estimation stage, the mapping network estimates S_0_ and R2* maps from the denoised images. Green box: the losses used for network training.

The architecture of the proposed CadamNet framework is illustrated in Figure 1. This cascade framework mainly consists of an image-denoising stage and a parameter estimation stage. Images at different TEs are concatenated and treated as a multi-channel 2D image input (analogous to the RGB channels in natural images). In the image denoising stage, a denoising network recovers high-quality T2*-weighted MR images from the corresponding noisy MR images, and then a mapping network maps the denoised T2*-weighted images to S_0_ and R2* parameter maps in the parameter estimation stage. The denoising network and mapping network were separately trained in an end-to-end manner. The loss function of the denoising network minimizes the pixel-wise differences between the denoised and reference noise-free images, which is defined as:

(1) $$L_{denoising}=\|\Phi \left(Y\right)-{X\|}_{2}^{2},$$

where Y are the noisy input T2*-weighted images, Φ is the denoising network, X is the reference noise-free images, and $\|\;{\|}_{2}$ denotes the *l*_2_ norm. The denoising loss enforces the denoising network to find the original signal from the noisy data. For the mapping network, the mapping loss is designed as follows:

(2) $$L_{mapping}=\|M\left(U\left(X_{denoise}\right)\right)-{X\|}_{2}^{2},$$

where $X_{denoise}=\Phi \left(Y\right)$ represent the denoised T2*-weighted images from the denoising network, **U** is the mapping network that computes the S_0_ and R2* maps given by the denoised T2*-weighted images, $X_{denoise}$, and **M** is the signal-decaying model for T2* relaxometry [16], which is given by

(3) $${\tilde{S}}_{i}=M\left(S_{0},R_{2}^{*},TE_{i}\right)={\tilde{S}}_{0}\cdot exp\left(-TE_{i}\cdot {\tilde{R}}_{2}^{*}\right),$$

where ${\tilde{S}}_{0}$ and ${\tilde{R}}_{2}^{*}$ are estimated parameter maps, and $TE_{i}$ denotes the *i*th TE for acquiring an MRI image ${\tilde{S}}_{i}$. The mapping loss, $L_{mapping}$, ensures that T2*-weighted images synthesized from the estimated parameter maps match the reference noise-free images.

Once the training process is completed, the noisy T2*-weighted images at multiple TEs can be efficiently converted to their corresponding parameter maps (${\tilde{S}}_{0}$, ${\tilde{R}}_{2}^{*}$) as follows:

(4) $$\left({\tilde{S}}_{0},\;{\tilde{R}}_{2}^{*}\right)=U\left(\Phi \left(Y\right)\right).$$

**Figure 2:**
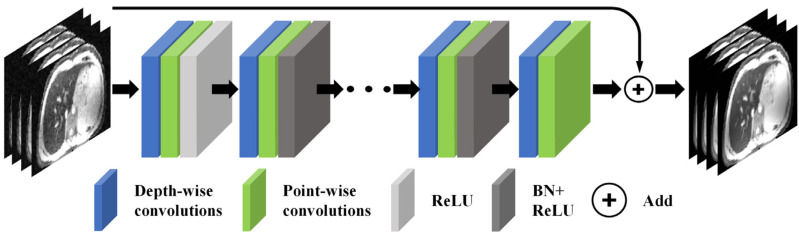
The architecture of denoising network.

The architecture of the denoising network is illustrated in Figure 2. A separable convolutional neural network (CNN) structure, which was proposed for application to hyperspectral image restoration [28], was adopted for image denoising. It has been shown that separable CNN can extract more delicate features of the images by separately considering the information of the position and channel. The key idea is to replace a standard convolution with two separable convolutions in spatial and channel (temporal here) dimensions. Convolution in the spatial dimension was independently performed in each channel (spatial convolution), while convolution in the channel dimension was performed using a 1D kernel (temporal convolution). The denoising network was composed of eight separable convolution layers. Each separable convolution layer contained 10 spatial convolution kernels with a size of 3 × 3 × 1 followed by 40 temporal convolution kernels with a size of 1 × 1 × 10, batch normalization, and ReLU [29]. The denoising network further used a residual learning strategy to improve training efficacy and efficiency [22].

**Figure 3:**
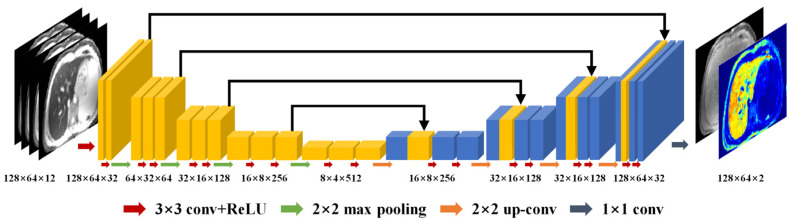
The architecture of the mapping network. The yellow and blue blocks are feature maps in the encoder and decoder, respectively.

As shown in Figure 3, the mapping network has the same structure as the popular 2D U-Net [30] architecture but with a 12-channel input for the mGRE data and 2-channel output for computed S_0_ and R2* maps. The U-Net had a symmetric structure and consisted of an encoder network and a decoder network. The encoder extracts robust and spatial invariant image features from the input images, while the decoder restores image details through multilevel deconvolution. Skip connections were used to concatenate features from the encoder to the decoder. The multi-scale structure of U-Net is effective in making use of global information in the images. When noise-free maps are taken as training targets, the mapping network will also have the function of noise reduction and can learn a mapping from noisy input to noise-free output.

### 2.4. Network Training

Both two-stage CadamNet and the single-stage mapping network (Figure 3) (denoted as MappingNet) were trained to demonstrate the performance of networks with and without the image denoising stage. Specifically, MappingNet outputs estimated parameter directly maps from the noisy T2*-weighted MR images. Both networks were trained on simulated datasets with specific noise levels (noise standard deviations ranging from 7 to 17 with an increment of two, and corresponding SNR ranging from 31 to 13) and also trained on the dataset with a mix of noise levels (referred to as CadamNet-m and MappingNet-m) [23]. The noise standard deviations of mixed noise levels range from 1 to 19 with a step of two (corresponding SNRs range from 216 to 11) and are evenly proportioned. The SNR of each dataset was calculated as the division of the mean intensity value within a selected ROI of liver parenchyma at the T2*-weighted image at the first TE by the noise’s standard deviation. The Adam algorithm was used to optimize the loss function, with an initial learning rate of 0.01, which was halved every dozen of epochs. The mini-batch size was set to 128. All networks were trained on a GeForce TITAN X Pascal GPU (NVIDIA Corporation, Santa Clara, CA, USA) and implemented in Python 3.6 with the Keras and a TensorFlow backend (Google, Mountain View, CA). Training times were approximately 12 h for the CadamNet/CadamNet-m and 10 h for the MappingNet/MappingNet-m.

### 2.5. Evaluation Metrics

The proposed CadamNet method was compared with MappingNet, as well as two conventional model-based methods (M^1^NCM [11] and PCANR [14]). It is worth noting that PCANR is the state-of-the-art method for liver R2* mapping. The normalized root-mean-square error (NRMSE) and structural similarity index (SSIM) [31] were employed to evaluate the performance of these R2* mapping methods. The NRMSE considers the pixel intensity-wise variations between reconstructed and reference R2* maps. The SSIM measures the structural similarity between reconstructed and reference R2* maps. The NRMSE and SSIM were computed inside the whole liver region (including liver parenchyma and vessels). The NRMSE is defined as:

(5) $$NRMSE=\frac{\|R_{2}^{*}-{\tilde{R}}_{2}^{*}\|{}_{2,\Omega }}{\|R_{2}^{*}\|{}_{2,\Omega }},$$

where $R_{2}^{*}$ and ${\tilde{R}}_{2}^{*}$ are the reference and estimated R2* values respectively, and $\|{\|\;}_{2,\Omega }$ denotes the *l*_2_ norm measured over the whole liver region Ω. The SSIM is defined as:

(6) $$SSIM=\frac{\left(2\mu_{x}\mu_{y}+C_{1}\right)\left(2\sigma_{xy}+C_{2}\right)}{\left(\mu_{x}^{2}+\mu_{y}^{2}+C_{1}\right)\left(\sigma_{x}^{2}+\sigma_{y}^{2}+C_{2}\right)},$$

where x and y are the reference and reconstructed R2* maps, $\mu_{x}$ and $\mu_{y}$ are the mean values of maps x and y, $\sigma_{x}$ and $\sigma_{y}$ are the standard deviation values of maps x and y, $\sigma_{xy}$ is the covariance of x and y, and $C_{1}={\left(k_{1}L\right)}^{2}$ and $C_{2}={\left(k_{2}L\right)}^{2}$ are variables that stabilize the division with weak denominator, where $k_{1}$ = 0.01, $k_{2}$ = 0.03 and *L* = max(x) − min(x).

### 2.6. Statistical Analysis

The SciPy Python package was used for statistical analysis. A Wilcoxon signed-rank test was performed to demonstrate the statistical difference between methods with a significance level of *p* < 0.05.

## 3. Results

### 3.1. Simulated Results

Table 1 presents the average NRMSE and SSIM measures of M^1^NCM, PCANR, MappingNet, CadamNet, MappingNet-m, and CadamNet-m on simulated testing datasets with varying noise levels (noise standard deviations σ_g_ ranging from 7 to 17 with an increment of two, and corresponding SNR ranging from 31 to 13). The selected noise levels are those that can be encountered in clinical practice. The SNR of each dataset is shown under the noise’s standard deviations in Table 1. The MappingNet and CadamNet models were trained on datasets under a fixed noise level and tested on datasets under a noise level identical to that in the training datasets. The performances of the deep learning-based methods in terms of NRMSE and SSIM were significantly superior (all *p* < 0.05) to the conventional M^1^NCM and PCANR algorithms. In all noise levels, CadamNet yielded better reconstruction performance (all *p* < 0.05) than all the other three methods. When the two models were trained on datasets with a mix of noise levels, CadamNet-m consistently outperformed (all *p* < 0.05) MappingNet-m under varying noise levels. In addition, CadamNet-m obtained NRMSE and SSIM scores comparable to those of CadamNet.

**Figure 4:**
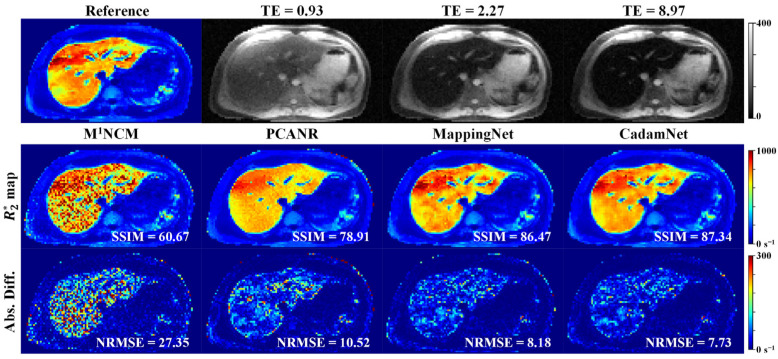
R2* maps for the simulated testing dataset with Rician noise (σ_g_ = 9). Top: Reference of R2* map and simulated noisy T2*-weighted images at three different echo times (ms). Bottom: R2* maps reconstructed using M^1^NCM, PCANR, MappingNet, and the proposed CadamNet methods, and the absolute difference maps are shown below the corresponding R2* maps. The SSIM of each R2* map is shown in its bottom right corner, and the NRMSE is shown in the absolute difference map.

**Figure 5:**
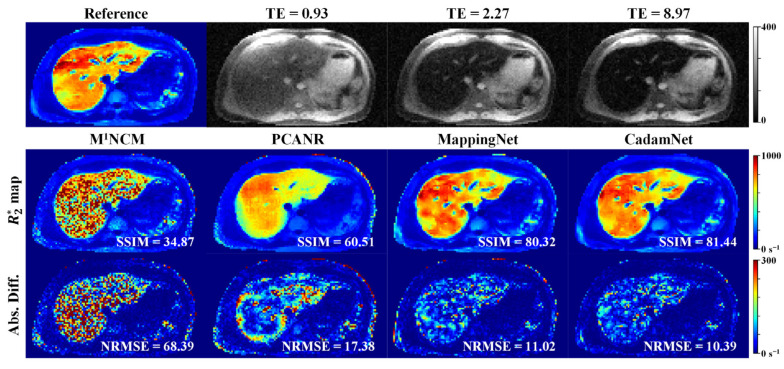
R2* maps for the simulated testing dataset with Rician noise (σ_g_ = 15). Top: Reference of R2* map and simulated noisy T2*-weighted images at three different echo times (ms). Bottom: R2* maps reconstructed using M^1^NCM, PCANR, MappingNet, and the proposed CadamNet methods, and the absolute difference maps are shown below the corresponding R2* maps. The SSIM of each R2* map is shown in its bottom right corner, and the NRMSE is shown in the absolute difference map.

Figure 4 and Figure 5 provide a visual evaluation of the results for one representative simulated testing dataset with noise standard deviations of 9 and 15, respectively. The R2* maps generated by the pixel-wise fitting using the M^1^NCM model were severely degraded by the noise. The effect of noise was well suppressed by the PCANR, MappingNet, and CadamNet methods. Although some small details at the right lobe of the liver became blurred (e.g., the R2* values of the regions with extremely high iron deposition were slightly underestimated), the CadamNet obtained the most consistent results with respect to the reference R2* map. The quantitative results from different methods suggest that CadamNet achieved the best performance in terms of SSIM and NRMSE.

**Figure 6:**
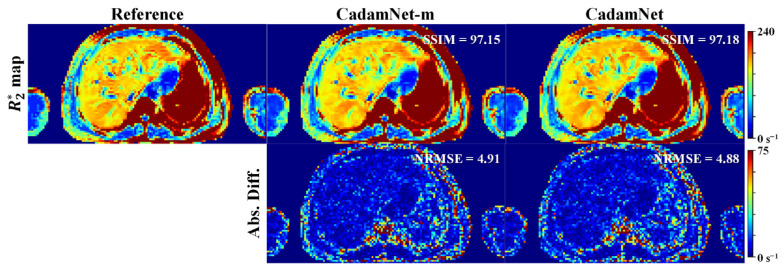
R2* maps reconstructed by CadamNet and CadamNet-m for the simulated testing dataset with Rician noise (σ_g_ = 17). It should be noted that CadamNet was trained by the simulated dataset with a noise standard deviation of 17, while CadamNet-m was trained by the simulated dataset with a mix of noise levels.

Figure 6 shows the R2* maps generated by CadamNet and CadamNet-m for one representative simulated testing dataset with a noise standard deviation of 17. CadamNet was trained using the simulated dataset with a noise standard deviation of 17, and CadamNet-m was trained using the simulated dataset with a mix of noise levels. Both models provided R2* maps close to the reference. Quantitatively, these two models produced close NRMSE and SSIM values.

**Figure 7:**
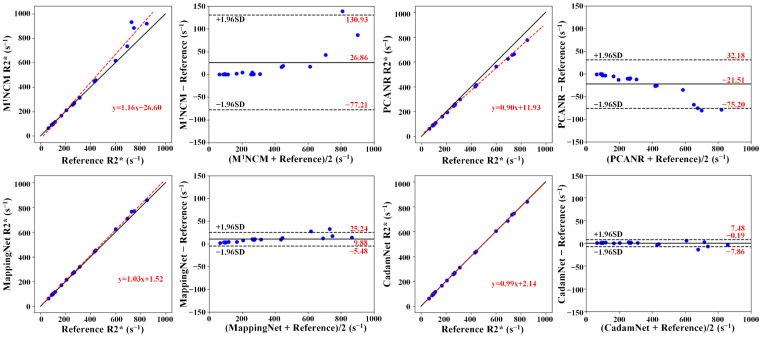
Scatterplots and Bland–Altman plots for the agreement of the mean R2* values in liver parenchyma (excluding vasculature) between the reference and the R2* maps reconstructed from different methods. The blue dots denote the subjects in the simulated testing liver dataset (σ_g_ = 17). The solid lines represent mean differences, and the dashed lines indicate 95% confidence intervals in Bland–Altman plots.

Scatterplots and Bland–Altman plots comparing the mean liver parenchyma R2* values of subjects in the simulated testing dataset under high noise levels (*σ*_g_ = 17) from different methods against the reference R2* values are illustrated in Figure 7. For low R2* values, all four methods produced accurate R2* estimates. For high R2* values, M^1^NCM overestimated the R2*, whereas PCANR underestimated the R2* values. Compared with M^1^NCM and PCANR, deep learning methods achieved a better agreement with the reference R2* values for liver parenchyma with narrower limits of agreement lines (the dashed lines), which were calculated at the ± 1.96*SD of the mean differences. CadamNet achieved further improved agreement compared with MappingNet. MappingNet yielded a mean difference of 9.88 ± 15.36 s^−1^, while CadamNet yielded a smaller mean difference of 0.19 ± 7.67 s^−1^. The mean liver parenchyma R2* value was obtained by averaging R2* values in all pixels of liver parenchyma. It should be noted that the liver parenchyma region (excluding vasculature) was manually segmented from the reference R2* map and applied to the R2* maps estimated by different methods.

### 3.2. Clinical Results

**Figure 8:**
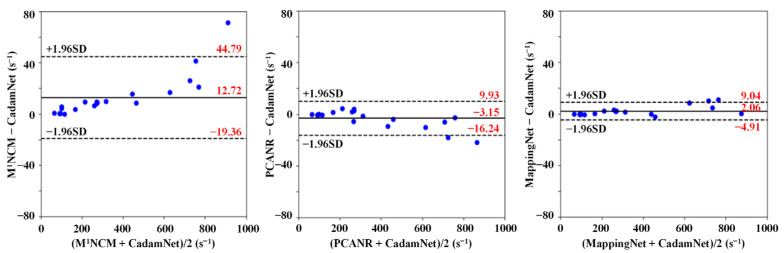
Bland–Altman analysis for the agreement of the mean R2* values in liver parenchyma (excluding vasculature) from different methods for clinical datasets. The blue dots denote the subjects in the clinical liver datasets. The solid lines represent mean differences, and the dashed lines indicate 95% confidence intervals.

Experiments were conducted on 21 real human liver data to verify the effectiveness of the proposed method on real clinical data. Figure 8 shows the results of Bland–Altman analysis for the agreement of the mean R2* values in liver parenchyma (excluding vasculature) between different methods. Due to the lack of knowledge of the exact noise levels in the in vivo data, CadamNet and MappingNet trained with a mix of noise levels were used for real clinical data. The approximate noise standard variances of these clinical data are in the regions from 6.0 to 12.0, which were calculated by multiplying the mean signal in a manually selected background ROI of all TE images with 0.80 [11]. For low R2*, these four methods agreed well. With regard to high R2*, M^1^NCM produced R2*s significantly higher than CadamNet, and PCANR produced lower R2*s than CadamNet. The R2*s from MappingNet were slightly higher than those from CadamNet. These results are consistent with those of the simulation study (Figure 7).

**Figure 9:**
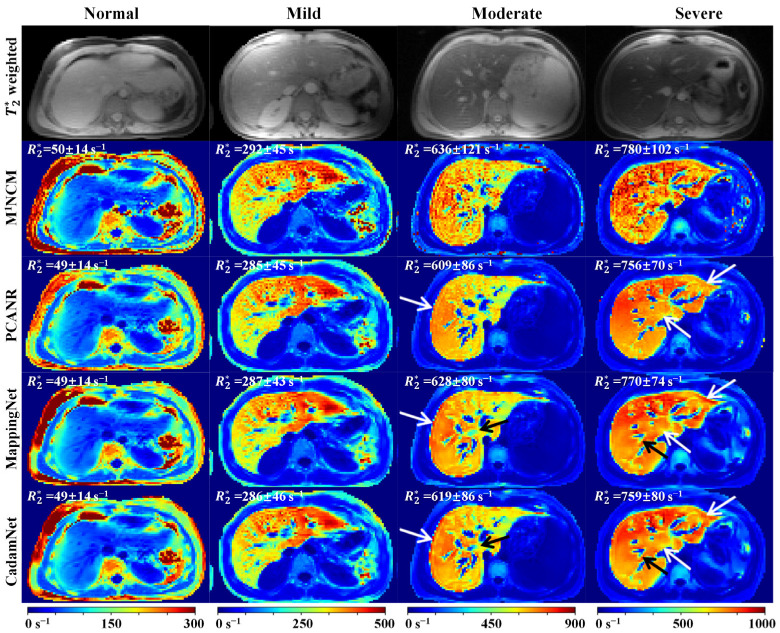
R2* maps for four representative real human liver data. The first row shows the T2*-weighted images at the first echo time (TE = 0.93 ms). The second-to-final rows show the R2* maps reconstructed using M^1^NCM, PCANR, MappingNet, and the proposed CadamNet methods. The mean and standard deviation of R2* values in liver parenchyma (excluding vasculature) are shown in the top-left corner of the R2* map.

Figure 9 shows the results of different methods on four representative clinical data from patients with normal, mild, moderate, and severe iron loads. For the normal liver, there was no apparent difference between the R2* maps produced by the four methods. For mild, moderate, and severe iron-overloaded livers, the R2* maps produced by the M^1^NCM were apparently degraded by the noise. The PCANR, MappingNet, and CadamNet methods all suppressed the noise effect and preserved tiny details. Compared with PCANR, deep learning-based methods achieved a better noise suppression effect in the regions, as pointed out by the white arrows. CadamNet slightly outperformed MappingNet in the regions, as pointed out by the black arrows, where MappingNet led to a blurring of tiny details. The mean R2* values of the liver parenchyma (excluding vasculature) produced by CadamNet were slightly lower than those of MappingNet for moderate and severe iron-loaded livers.

## 4. Discussion

In this work, we proposed a deep learning method called CadamNet for R2* mapping of an iron-loaded liver. CadamNet first employed a separable CNN to denoise the measured multi-echo T2*-weighted images and then employed a U-Net to estimate R2* map from the denoised data. The validation results on both simulated and clinical data demonstrated that the two-stage CadamNet method outperformed the single-stage U-Net mapping method and conventional model-based methods in the liver R2* mapping, especially under the condition of high noise level and in the presence of moderate to severe iron overload.

CadamNet can be considered a data-driven method for liver R2* mapping. Compared with conventional model-based methods (M^1^NCM and PCANR), CadamNet showed significantly improved reconstruction performance. The M^1^NCM method fitted the data in a pixel-wise manner, and this resulted in noisy maps because of the lack of utilization of neighborhood information [32]. PCANR suppressed the noisy appearance in the R2* maps but produced oversmoothing in certain liver parenchyma regions under high noise levels. The suboptimal performance of PCANR in certain regions may be due to the fact that this algorithm cannot find effective temporal signals with similar underlying R2* values for regularization, especially at high noise levels. The results showed that CadamNet could simultaneously suppress the noise and preserve details in R2* maps. This might be attributed to the fact that the CNN-based architecture of CadamNet can leverage the prior spatial patterns of the signals in the images to reduce the effect of noise.

Both CadamNet and MappingNet can reduce the effect of noise on the liver R2* mapping because these two networks were trained to approximate a mapping from noisy serial image patches to noise-free reference image patches synthesized from parameter maps. Compared with pixel-based training [33], patch-based training can exploit neighboring information and prior spatial patterns in images for noise reduction. CadamNet slightly outperformed MappingNet in terms of NRMSE and SSIM. CadamNet and MappingNet have nearly the same number of trainable parameters (CadamNet contained 8,766,254 parameters and MappingNet 8,633,122 parameters). The performance gap could not be attributed to a difference in network capacity. Thus, the improved performance of two-state CadamNet over single-stage Mapping net is related to the incorporation of the auxiliary denoising task. The image denoising stage tries to find the real signal from the noisy images, and it will reduce the disruption of the noise in parameter estimation and simplify the subsequent parameter mapping problem.

The performance of deep learning-based denoising methods is related to noise levels [23]. The noise level in the clinical MR images is generally unknown. Thus, it is important to evaluate the performance of CadamNet on data with unknown noise levels. In this study, we trained CadamNet using data with a mix of noise levels and evaluated its performance on both simulations with varying noise levels and clinical data. The results demonstrated that CadamNet trained with mixed noise consistently achieved performance comparable to that of CadamNet trained using data with a specific noise level. This means that training CadamNet using data with a mix of noise levels may be one of the potential solutions for real clinical data applications without prior knowledge of the noise levels.

With respect to the computational cost, CadamNet was proven to be a time-efficient method. Although the training of the network requires many hours, the prediction of a single-slice liver R2* map can be accomplished within ~0.1 s (using a GeForce TITAN X Pascal GPU). Compared to conventional model-based liver R2* mapping approaches that generally take approximately 1 min (implemented using Python on a modern PC with four cores), the proposed method achieves approximately one magnitude of acceleration.

Our current work has several limitations. First, the trained CadamNet model can only be applied to multi-echo images with TE patterns identical to the training data. TE sampling patterns in R2* relaxometry sequences usually vary in different scanners and/or centers. For the application to R2* relaxometry in practice, CadamNet needs to be retrained using data synthesized with actual TEs. Another possible solution is to incorporate the TE information into the input and learn a TE-independent model. Second, the performance of CadamNet was only demonstrated on datasets with spatially stationary Rician noise. For single-coil acquisition or SENSE [34] reconstructed images from array coils, the magnitude signal can be considered Rician distributed. However, for a multiple-coil acquisition with sum-of-squares or GRAPPA [35] reconstruction, the magnitude signal follows a non-central chi distribution. The variance of noise becomes position-dependent in images reconstructed by using parallel MRI. The extension of CadamNet to spatially variable non-central chi noise is expected in the future. Although complex data is not always available and may be corrupted by phase errors, complex R2* fitting does not suffer bias caused by the nonzero mean noise and can be used to address background field variation effects in R2* mapping [36,37]. Third, although the fat-suppression technique is a solution to eliminate most of the lipid signal [38], another way to correct fat-water modulations is to model the T2* decay and fat interactions simultaneously [39]. Finally, although we used separate CNN and U-Net as the backbone networks of CadamNet, which have been validated in denoising and parameter mapping tasks, respectively, the choice of backbone network is unrestricted. More sophisticated network architectures, such as ConvNeXt [40] and Swin Transformer [41], may be adopted instead to improve the performance of CadamNet in future work. Physical constraints and experiential regularizations in conventional methods could be incorporated into the CadamNet framework, in a way similar to the recently developed unrolling techniques [42,43,44,45], for further improving reconstruction performance.

## 5. Conclusions

In conclusion, this work presents a novel deep learning method for liver R2* mapping. The proposed two-stage CadamNet method reduces the noise effect on R2* mapping by implementing a denoising network and a mapping network in a sequential manner. Extensive simulated datasets constructed from clinical liver data are used to train the networks. CadamNet demonstrated improved accuracy and efficiency over the single-stage mapping network and conventional model-based fitting methods. The cascade framework of CadamNet is expected to be useful for quantitative MR applications involving T1 relaxometry, diffusion, and perfusion parameter mapping.

## Figures and Tables

**Table 1:** The NRMSE (%) and SSIM (%) measures of different methods on simulated testing datasets with various noise standard deviations (σ_g_).

Methods	Metrics	σ_g_ = 7 (SNR = 30.95)	9 (SNR = 24.07)	11 (SNR = 19.71)	13 (SNR = 16.64)	15 (SNR = 14.46)	17 (SNR = 12.70)
M^1^NCM	NRMSE SSIM	6.15 (4.00) 94.03 (7.46)	8.49 (6.26) 91.40 (10.14)	10.55 (7.69) 88.22 (12.32)	12.86 (9.98) 85.34 (14.66)	15.30 (12.14) 82.33 (16.66)	18.03 (13.81) 78.48 (18.71)
PCANR	NRMSE SSIM	5.91 (2.19) 94.55 (4.63)	7.00 (2.44) 92.68 (5.85)	7.82 (2.60) 91.12 (6.73)	8.69 (2.91) 89.18 (8.07)	9.49 (2.96) 87.81 (8.49)	10.24 (3.09) 85.91 (9.57)
MappingNet	NRMSE SSIM	4.06 (1.73) 97.40 (3.19)	4.87 (2.00) 96.35 (3.47)	5.71 (2.21) 95.50 (3.97)	6.35 (2.36) 94.63 (4.28)	6.96 (2.56) 93.53 (5.21)	8.50 (2.49) 92.40 (5.96)
CadamNet	NRMSE SSIM	3.96 (1.66) 97.60 (2.33)	4.73 (1.87) 96.64 (3.16)	5.34 (2.02) 96.05 (3.29)	6.00 (2.21) 95.00 (3.86)	6.69 (2.39) 94.03 (4.82)	7.25 (2.50) 93.09 (5.30)
MappingNet-m	NRMSE SSIM	4.20 (1.88) 97.26 (2.78)	4.93 (2.04) 96.29 (3.61)	5.60 (2.23) 95.49 (4.05)	6.25 (2.31) 94.60 (4.50)	6.97 (2.50) 93.46 (5.31)	7.57 (2.73) 92.19 (6.33)
CadamNet-m	NRMSE SSIM	4.06 (1.76) 97.48 (2.41)	4.77 (1.98) 96.55 (3.23)	5.43 (2.11) 95.79 (3.66)	6.05 (2.20) 95.02 (3.96)	6.68 (2.36) 94.05 (4.64)	7.22 (2.51) 93.00 (5.34)

Notes: Results are shown as the mean (SD) values over the testing datasets.

## Data Availability

The source code in this study is available upon reasonable request from the corresponding author.
